# Anti-HBs Seroprevalence in Blood Donors from Tyrol, Austria

**DOI:** 10.3390/vaccines12101156

**Published:** 2024-10-11

**Authors:** Lisa Seekircher, Annelies Mühlbacher, Lena Tschiderer, Gregor A. Wachter, Manfred Astl, Harald Schennach, Anita Siller, Peter Willeit

**Affiliations:** 1 Institute of Clinical Epidemiology, Public Health, Health Economics, Medical Statistics and Informatics, Medical University of Innsbruck, 6020 Innsbruck, Austria; 2 Central Institute for Blood Transfusion and Immunology, University Hospital Innsbruck, Tirol Kliniken GmbH, 6020 Innsbruck, Austria; 3 Ignaz Semmelweis Institute, Interuniversity Institute for Infection Research, 1090 Vienna, Austria; 4 Department of Public Health and Primary Care, University of Cambridge, Cambridge CB1 8RN, UK

**Keywords:** antibodies against the hepatitis B surface antigen, hepatitis B virus, blood donors, seroprevalence, antibody levels, HBV vaccination

## Abstract

**Background/Objectives:** Antibodies against the hepatitis B surface antigen (anti-HBs) are a marker of immunity against hepatitis B virus (HBV) infections. There is uncertainty about the anti-HBs seroprevalence in the general population of Austria. **Methods**: We conducted a cross-sectional analysis in blood donors from the Federal State of Tyrol in Austria (August–September 2023) to estimate anti-HBs seroprevalence and median antibody levels. **Results**: We enrolled 3935 blood donors (median age 47.6 years [25th–75th percentile: 33.3–56.6]; 40.7% female), who were hepatitis B surface antigen negative and had no detectable HBV-DNA. Overall seroprevalence was 51.4% (95% CI: 49.8–52.9%). Anti-HBs seropositivity decreased with higher age (*p* < 0.001), with 70.3% (66.1–74.3%) being seropositive among participants < 25 years of age and 30.2% (24.2–36.9%) in those aged ≥ 65 years. More females than males were seropositive (54.3% [51.8–56.7%] vs. 49.4% [47.4–51.4%]; *p* = 0.003). Seroprevalence was significantly higher in urban than in rural areas in participants aged 40 to <55 (*p* = 0.045) and ≥55 years (*p* = 0.001). Among 2022 seropositive participants, the overall median anti-HBs antibody level was 539.3 IU/L (25th–75th percentile: 116.3–5417.0). Furthermore, 5% of the participants had an anti-HBs antibody level between 10 and <20 IU/L, 18% between 20 and <100 IU/L, and 77% ≥100 IU/L. **Conclusions**: Anti-HBs seroprevalence in blood donors from Tyrol, Austria, was 51.4% between August and September 2023 and differed across age, sex, and residence area. Catch-up vaccination programs, especially targeting the elderly living in rural areas, are needed to close HBV immunity gaps.

## 1. Introduction

Hepatitis B virus (HBV) is a highly infectious pathogen primarily targeting the liver, leading to inflammation known as hepatitis. Disease severity can range from asymptomatic to severe symptoms. Acute HBV infection can become chronic if it persists for more than six months, leading to long-term health risks, including liver failure and cancer [1,2]. While about 5% of adults with hepatitis develop a chronic course of the disease, the proportion of cases in infants and children is up to 90% [3]. In 2022, about 254 million people were living with a chronic HBV infection, and about 1.1 million deaths worldwide were caused by HBV-related diseases [4]. The prevalence of chronic HBV infections varies widely between countries, with the highest rates in the African region (5.8%) and the Western Pacific region (5.0%), followed by the South East Asia Region (3.0%) and the Eastern Mediterranean Region (2.1%) [4]. However, the prevalence of chronic HBV is much lower in the European Region (1.2%) and the Region of the Americas (0.5%). Despite the major efforts of the World Health Organization (WHO) to control HBV by 2030 and achieve a 90% reduction in new chronic infections and a 65% reduction in mortality, hepatitis B remains a substantial public health problem [5]. In data from 187 countries, the estimated number of deaths caused by viral hepatitis has risen from 1.1 million individuals to 1.3 million individuals in 2022, of which 83% were caused by HBV [4].

HBV is transmitted through contact with body fluids, with a particularly high risk of contracting HBV through blood. In Europe, HBV is primarily transmitted among adults through sexual contacts or unsafe injections, but HBV can also be transmitted during birth if the mother is infected, which is one of the main causes of transmission in poorly developed countries. Before 1970, HBV was also transmitted through blood transfusions [5]. With the introduction of systematic HBV screenings for all blood donations, transfusion-related hepatitis B has significantly decreased in many countries. In Austria, all blood donors are routinely tested for hepatitis B surface antigen (HBsAg) and HBV-DNA, and individuals with a known HBV infection are permanently excluded from blood donation. In contrast to neighboring countries such as, for example, Germany, testing for anti-hepatitis B core antigen (HBc) in order to detect donors that have gone through an HBV infection in the past is not mandatory for blood donors in Austria. However, individuals that have knowingly gone through an HBV infection are deferred permanently from blood donation in Austria.

The most important prevention measure against HBV infection is the HBV vaccine, which stimulates the immune system to produce antibodies directed against HBsAg (anti-HBs). In 1992, the WHO recommended implementing universal hepatitis B vaccination in national immunization schedules [3]. In Austria, the vaccine has been part of the free child vaccination program since 1998, offering complimentary HBV vaccination to all children residing in Austria up to the age of 15 [6,7]. Further, in Austria, all newborns of infected mothers that are HbsAg positive are passively immunized and vaccinated within twelve hours of birth to prevent transmission of HBV infection. Monitoring anti-HBs antibody levels in vaccinated individuals helps assess immune status and identify potential immunity gaps in the general population. Anti-HBs antibody levels of 10 IU/L or higher are generally considered protective [8,9]. Lower or undetectable levels may indicate a need for booster vaccination, particularly in high-risk populations, but based on randomized clinical trials, there is no scientific evidence that supports the need for booster doses in healthy individuals previously vaccinated with the hepatitis B vaccine [10]. However, data on anti-HBs seroprevalence and/or anti-HBs antibody levels in the Austrian population are scarce, highlighting the importance of performing the present study in order to directly measure if vaccination initiatives that were set up in the past lead to satisfactory protection of the Austrian population.

The aim of this study was to report on (i) the proportion of seropositive individuals, i.e., anti-HBs antibody levels ≥ 10 IU/L, as an indicator of collective immunity against HBV and (ii) the characteristics of individuals without protective antibody levels to identify potential gaps of immunity in Tyrol, Austria, using data obtained from a blood donor cohort.

## 2. Materials and Methods

We conducted a cross-sectional analysis of blood donors in the Federal State of Tyrol in Austria. Participants were recruited between 16 August 2023 and 25 September 2023 at 36 blood donation events across all districts of Tyrol, except the district of Lienz. Individuals were eligible for inclusion if they (i) were aged between 18 and 70 years, (ii) were permanent residents in Tyrol, and (iii) fulfilled the general requirements for donating blood. Individuals who report to have had an HBV infection are permanently deferred from blood donation. Out of 4699 eligible individuals, 3935 participated in the study, corresponding to a participation rate of 83.7%. The study was approved by the Ethics Committee of the Medical University of Innsbruck (no. 1032/2024).

Serum samples were drawn at blood donation events, kept at room temperature until the end of the donation event, and then immediately shipped to the laboratory of the Central Institute for Blood Transfusion and Immunology of the University Hospital in Innsbruck, Austria. Upon arrival at the laboratory, samples were stored at 4 °C until analysis. Samples were screened for HBV status using three different assays. First, samples were qualitatively assessed for HBsAg using the Abbott HBsAg Next Qualitative Reagent Kit chemiluminescent microparticle immunoassay analyzed on the Alinity i or the Alinity ci instrument (Abbott Ireland, Sligo, Ireland) [11]. According to the manufacturer, this assay has a sensitivity of 100% (95% confidence interval [CI]: 99.17–100%) and a specificity of 99.96% (99.87–99.99%). Second, samples were qualitatively assessed for HBV-DNA using the PCR-Kit PoET HBV on the PoET instrument (GFE, Frankfurt/Main, Germany) at the blood donor service of the Bavarian Red Cross, Wiesentheid, Germany [12]. According to the manufacturer, this assay has a 95% detection limit of 1.6 IU/mL (95% CI: 1.4–2.1) and a specificity of 100%. Third, we quantitatively determined anti-HBs antibodies using the Roche Elecsys^®^ Anti-HBS II electrochemiluminescent immunoassay on the Cobas e 601 analyzer (Roche, Mannheim, Germany) [9]. The measuring range of this assay ranges from 2.00 to 100,000 IU/L, and values below the limit of detection are reported as <2.00 IU/L. Samples with an anti-HBs antibody value > 1000 IU/L were automatically diluted by the analyzer at a ratio of 1:100, and concentrations were calculated by multiplying the result by the dilution factor. Following the manufacturer manual, anti-HBs antibody values < 10 IU/L were considered non-reactive, indicating seronegativity. Anti-HBs antibody values ≥ 10 IU/L were considered as reactive, indicating seropositivity, which was shown to have a sensitivity of 100% in both anti-HBs positive samples of vaccines (*n* = 296) and recovered individuals from a HBV infection (*n* = 373) and specificities of 99.78% in anti-HBs negative samples of blood donors (*n* = 2673) and 99.45% in routine anti-HBs negative samples (*n* = 1623) [9]. Within-run and between-run precision on the Cobas e 601 analyzer was determined by the manufacturer in five different human serum samples with two runs per day in duplicate each for 21 days (*n* = 84) [9]. The coefficient of variation for within-run precision (repeatability) ranged from 1.7% to 4.7% and for between-run precision (intermediate precision) from 3.4% to 6.8% [9]. We did not perform tests on anti-HBc antibodies in the present study.

Continuous variables were described using medians and 25th to 75th percentiles, while categorical variables were presented with numbers and percentages. Seroprevalence for anti-HBs, i.e., anti-HBs antibody values ≥ 10 IU/L, was quantified and reported with Agresti–Coull 95% CIs [13], and anti-HBs antibody levels were summarized as medians and 25th to 75th percentiles. We also reported the proportions of individuals within the anti-HBs categories 10 to <20, 20 to <100, and ≥100 IU/L. We chose these categories because, according to the Austrian Vaccination Plan 2023/2024 [7], different titer checks and booster vaccinations are recommended for at-risk groups, depending on which of these anti-HBs categories they belong to. Differences in anti-HBs seroprevalence and antibody levels (among seropositive individuals) were analyzed by age group (≤25, 25 to <30, 30 to <35, 35 to <40, 40 to <45, 45 to <50, 50 to <55, 55 to <60, 60 to <65, and ≥65 years) and sex (female and male). Additionally, across age groups (<25, 25 to <40, 40 to <55, ≥55), we quantified the percentage of seropositive individuals by prior pregnancy status (yes and no), residence area (urban and rural), and residence district in the Federal State of Tyrol (Innsbruck-Stadt, Innsbruck-Land, Schwaz, Kufstein Kitzbühel, Imst, Landeck, and Reutte). We tested for differences across subgroups in anti-HBs seroprevalence using logistic regression and in anti-HBs antibody levels using the Mann–Whitney U test or the Kruskal–Wallis test. *p* values ≤ 0.05 were deemed statistically significant. Analyses were performed using Stata 15.1.

## 3. Results

The results are reported in accordance with the Strengthening the Reporting of Observational Studies in Epidemiology (STROBE) guidelines (Supplementary Materials Table S1) [14].

In total, 3935 blood donors were enrolled in our study. The characteristics of the study participants are summarized in Table 1**.** Median age was 47.6 years (25th to 75th percentile: 33.3–56.6), 40.7% were female, and 66.8% of females reported a prior pregnancy. 40.7% of the participants lived in urban areas. The majority were from the districts of Kufstein (26.8%), Landeck (16.0%), and Innsbruck-Land (14.6%).

**Figure 1:**
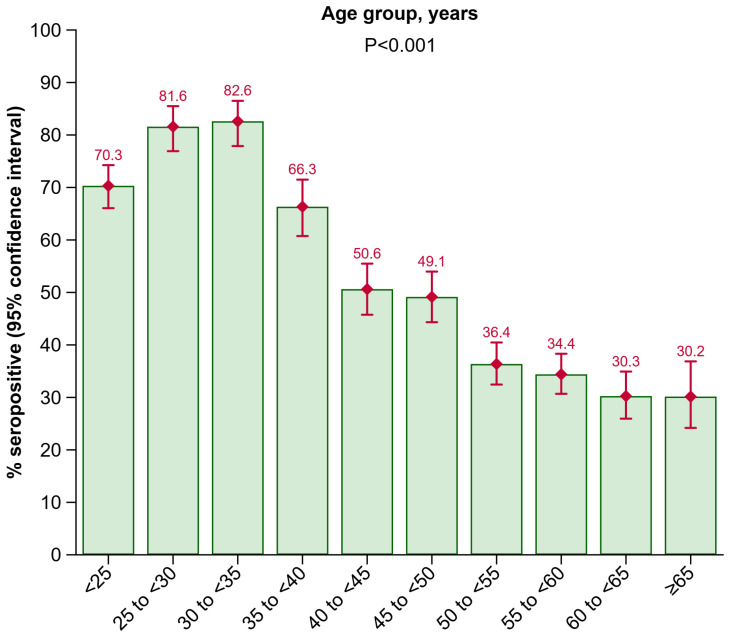
Seroprevalence of anti-HBs by age group. Abbreviations: Anti-HBs, antibody to hepatitis B surface antigen.

In the HBV screening, all 3935 participants were HBsAG negative, and all 3671 participants who underwent a HBV-DNA-PCR test had no detectable HBV-DNA. Overall, 2022 participants (51.4% [95% CI: 49.8–52.9%]) had anti-HBs antibody levels ≥ 10 IU/L, i.e., were seropositive. Anti-HBc antibodies were not tested in the present study. Figure 1 summarizes the seroprevalence of anti-HBs by age group. The odds of being anti-HBs positive decreased with higher age (*p* < 0.001). Positivity rates were 70.3% (66.1–74.3%) in the <25 years age group, rising to 81.6% (76.9–85.5%) in the 25 to <30 years age group, and peaking to 82.6% (77.9–86.5%) in the 30 to <35 years age group. In older age groups, seroprevalence declined with 66.3% (60.7–71.5%) in the 35 to <40 years age group, 50.6% (45.7–55.5%) in the 40 to <45 years age group, 49.1% (44.3–54.0%) in the 45 to <50 years age group, 36.4% (32.4–40.5%) in the 50 to <55 years age group, 34.4% (30.7–38.3%) in the 55 to <60 years age group, 30.3% (26.0–34.9%) in the 60 to <65 years age group, and 30.2% (24.2–36.9%) in the ≥65 years age group.

**Figure 2:**
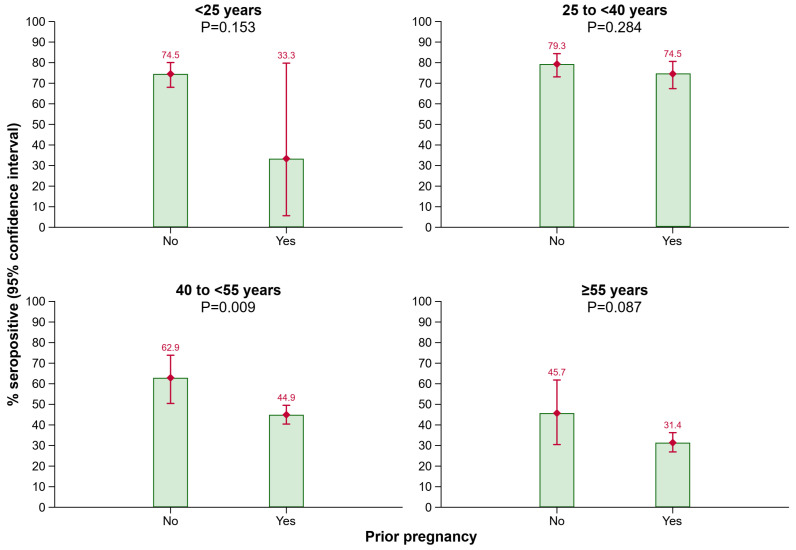
Seroprevalence of anti-HBs by prior pregnancy in different age groups. Abbreviations: Anti-HBs, antibody to hepatitis B surface antigen.

Furthermore, more females than males had anti-HBs antibody levels ≥ 10 IU/L (54.3% [51.8–56.7%] vs. 49.4% [47.4–51.4%]; *p* = 0.003). In the female 40 to <55 years age group, more individuals without a prior pregnancy than with a prior pregnancy were seropositive (62.9% [50.4–73.9%] vs. 44.9% [40.4–49.5%]; *p* = 0.009). Among the other age groups, no significant difference in anti-HBs positivity rates regarding the pregnancy status was detected; however, results from the <25 years age group need to be interpreted with caution as only a very limited number of persons were aged < 25 and reported a prior pregnancy (Figure 2).

**Figure 3:**
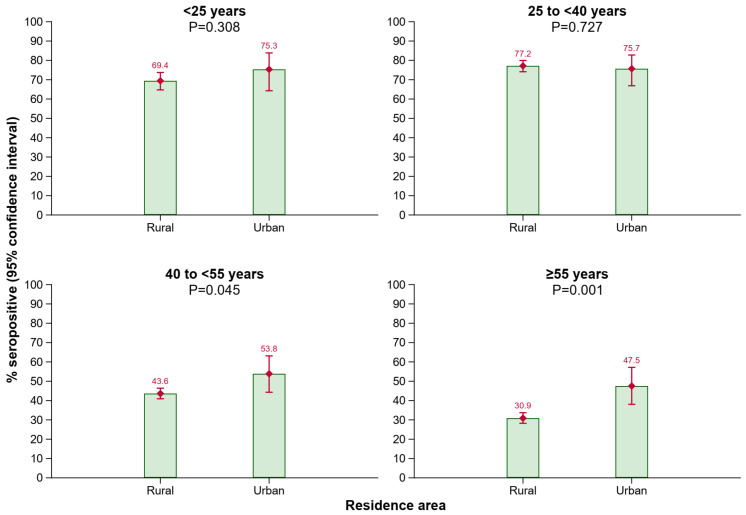
Seroprevalence of anti-HBs by residence area in different age groups. Abbreviations: Anti-HBs, antibody to hepatitis B surface antigen.

As shown in Figure 3, a lower proportion of individuals in older age groups living in rural areas of Tyrol had anti-HBs antibody levels ≥ 10 IU/L compared to those living in urban areas of Tyrol (age group 40 to <55 years: *p* = 0.045; age group ≥ 55 years: *p* = 0.001).

**Figure 4:**
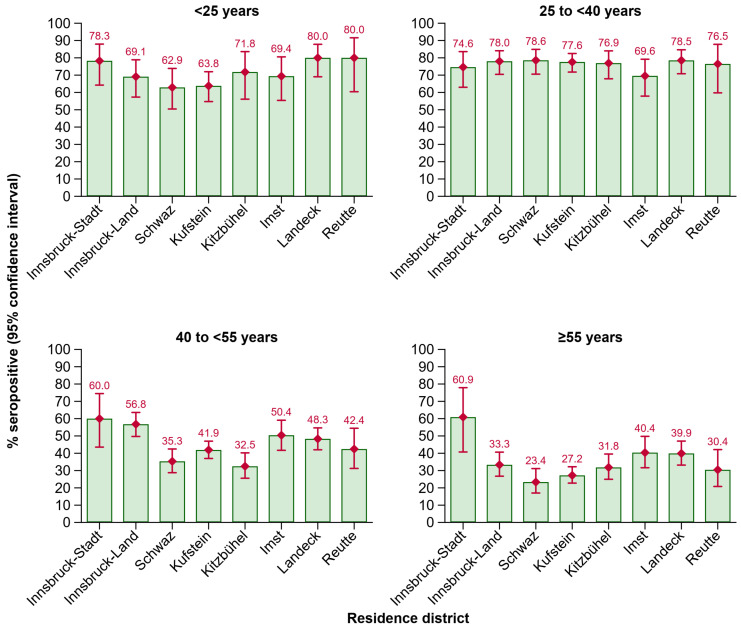
Seroprevalence of anti-HBs by residence district in different age groups. Abbreviations: Anti-HBs, antibody to hepatitis B surface antigen.

In these age groups, seropositivity also fluctuated across the districts of Tyrol (Figure 4). In the 40 to <55 years age group, the positivity rate was significantly lower in the districts of Schwaz (*p* = 0.008) and Kitzbühel (*p* = 0.003) as compared to Innsbruck-Stadt. In the ≥55 years age group, seropositivity was lower in individuals living in the districts of Innsbruck-Land (*p* = 0.013), Schwaz (*p* = 0.001), Kufstein (*p* = 0.001), Kitzbühel (*p* = 0.009), and Reutte (*p* = 0.011), when compared to Innsbruck-Stadt.

**Figure 5:**
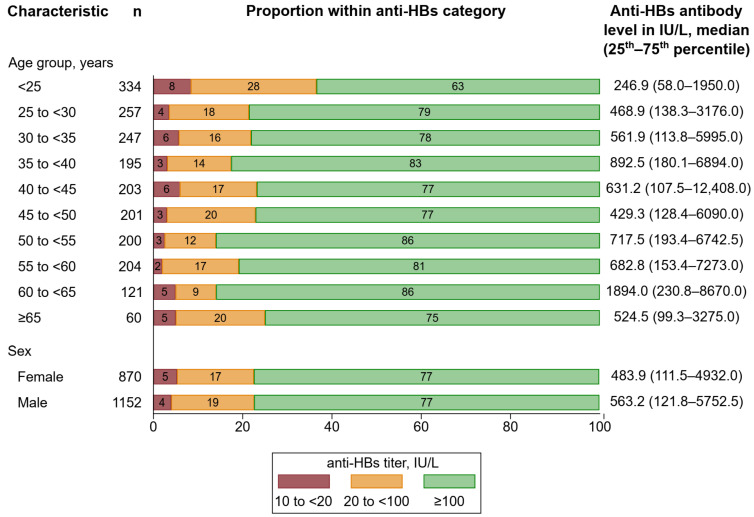
Distribution of anti-HBs levels by age group and sex. Percentages may not sum up to 100 due to rounding. Abbreviations: Anti-HBs, antibody to hepatitis B surface antigen.

Among 2022 seropositive participants, the overall median anti-HBs antibody level was 539.3 IU/L (25th to 75th percentile: 116.3–5417.0). Furthermore, 5% of the participants had an anti-HBs antibody level between 10 and <20 IU/L, 18% between 20 and <100 IU/L, and 77% ≥ 100 IU/L. Figure 5 depicts proportions of participants within anti-HBs categories 10 to <20, 20 to <100, and ≥100 IU/L by age group and sex. Anti-HBs antibody levels differed across age groups (*p* < 0.001). Nevertheless, the proportions of participants within anti-HBs categories appeared to be similar across age groups. Among individuals aged ≥ 25, the proportion of participants within the category ≥ 100 IU/L ranged from 75% to 86%. However, among individuals aged < 25 years, the proportion of participants within the category ≥ 100 IU/L appeared to be lower with 63%. We detected no difference in anti-HBs antibody levels by sex (*p* = 0.152), and proportions of participants within anti-HBs categories were highly similar.

## 4. Discussion

The present study reports on the anti-HBs seroprevalence and anti-HBs antibody levels among 3935 blood donors from the Federal State of Tyrol, Austria, between August and September 2023. Overall, 51.4% (95% CI: 49.8–52.9%) had anti-HBs antibody levels ≥ 10 IU/L and are therefore considered to be protected from HBV infection. The odds of being anti-HBs positive decreased with higher age and were lower in males than in females. Furthermore, in older age groups, anti-HBs positivity rates fluctuated across the districts of Tyrol and were lower in individuals living in rural areas compared to those in urban areas. Among seropositive participants, the proportions of participants within anti-HBs categories of 10 to <20, 20 to <100, and ≥100 IU/L were similar between females and males and generally across age groups. However, in the age group < 25 years, fewer participants were in the ≥100 IU/L category when compared to the other age groups.

To the best of our knowledge, this is the first large-scale study to assess seroprevalence and levels of anti-HBs antibodies in Austria. Our analysis uncovered that more than half of the participants (51.4%; 95% CI: 49.8–52.9%) had anti-HBs antibody levels ≥ 10 IU/L, which is considered to be protective. In relation to that, anti-HBs antibodies were also analyzed in cohorts from other countries in the past 5 years, indicating similar overall anti-HBs seroprevalences, with 53.9% in Serbia [15], 44.1% in Argentina [16], 52.9% in China [17], 34% in Vietnam [18], but only 3.34% in Saudi Arabia [19]. These data highlight that, especially in Europe/America, anti-HBs seroprevalence is rather high and similar among countries but is strikingly lower in Saudi Arabia, in line with the higher prevalence of chornic HBV infections in the Eastern Mediterranean Region (2.1%) compared to the European Region (1.2%) and the Region of the Americas (0.5%) [4]. However, when we compare the data from Serbia in more detail with our study, individuals aged 20–59 years were seropositive in 17.7% (15.5–19.9%) in Serbia, whereas in our study seroprositivity was much higher (36.4% in the 50 to <55 age group and 34.4% in the 55 to <60 age group). This can be potentially explained by more dense vaccination campaigns in adults in Austria compared to Serbia. Anti-HBs seroprevalence studies were also performed in other countries more than 10 years ago, with, e.g., 23.8% in Italy [20], 28.6% in Luxembourg [21], 24.4% in Croatia [22], 23.0% in Turkey [23], 27.7% in Laos [24], 15.9% in Egypt [25], and 2.5–12.7% in Nigeria [26,27]. All of these countries have implemented universal HBV immunization programs between the early 1990s and the mid-2000s. However, regional seroprevalence differences are likely due to varying HBV vaccination practices and infection rates. Furthermore, these studies were conducted in different years, ranging from 2006 to 2021, and involved study samples with diverse age groups, which makes it almost impossible to perform direct comparisons to our present study and to draw conclusions about to date protective HBV immunity in these countries. According to the Global Hepatitis Report 2024, Africa accounts for 63% of new HBV infections, while only 18% receive a HBV vaccination at birth [4]. Further, the western Pacific region accounts for 47% of HBV deaths, and access to HBV treatment is still low [4]. These data indicate that, especially in these regions, access to HBV birth doses as well as booster vaccinations needs to be improved in order to prevent HBV infections and HBV-associated deaths [4]. In contrast, in America as well as the European region, the prevalence of chronic HBV among the general population is much lower, showing that vaccination programs that were established many years ago are important to close gaps of immunity and to reduce HBV prevalent cases [4].

In Austria, HBV vaccination is an essential part of public healthcare to prevent HBV infections and their complications. Since 1998, the HBV vaccine has been part of the child vaccination program in Austria, providing all children living in Austria up to the age of 15 with free vaccination [6,28,29]. In 2001, a new vaccination scheme vaccinating toddlers concomitantly against six diseases, including hepatitis B, was implemented, leading to widespread immunization against hepatitis B [6,28]. In 2004 and 2005, an Austria-wide vaccination campaign against hepatitis A and B was carried out, with vaccinations at a reduced price [30]. Additionally, since 1985, the General Accident Insurance Institution (AUVA) has been conducting a HBV vaccination campaign for at-risk groups to prevent occupational HBV infections [31]. The National Vaccination Plan 2023/2024 [7] states as a primary immunization schedule a series of three doses, which can be administered as part of the six-in-one vaccine, as a monocomponent vaccine, or as a combination with vaccination against hepatitis A. After the primary immunization in infancy or early childhood, a booster vaccination is recommended between the ages of 7 and 15 years. Further booster vaccinations every 10 years are recommended for high-risk groups. These include individuals with an indication such as chronic liver disease, frequent need for plasma products, predialysis or dialysis, or existing immunodeficiency, as well as individuals who have multiple sexual partners, use intravenous drugs, travel to regions with high HBV prevalence, have a refugee and migration background or are involved in their care, or have a high occupational risk (e.g., healthcare workers, public safety workers, workers in blood banks and transfusion services, staff in care facilities, personnel in correctional facilities, funeral and mortuary workers, tattoo artists and body piercers, and workers in waste and disposal management). For individuals who missed the vaccination during childhood, it is recommended to receive the hepatitis B vaccine later in life. The hepatitis B vaccination can be given at any age and is generally recommended in Austria up to the age of 65.

In our study, the distribution of seropositive participants differed across age, sex, and residence area. Our analysis showed that anti-HBs positivity rates tended to decrease with age. We found the highest anti-HBs positivity rates in the age groups 25 to <30 years (81.6% [95% CI: 76.9–85.5%]) and 30 to <35 years (82.6% [77.9–86.5%]), followed by the age groups < 25 years (70.3% [66.1–74.3%]) and 35 to <40 (66.3% [60.7–71.5%]). These groups consist of participants born between 1998 and 1983 who were eligible for the free child vaccination program initiated in 1998. This suggests that initiatives promoting HBV vaccination in Austrian children effectively increased anti-HBs seropositivity. Other studies also reported a decreasing seroprevalence with higher age [15,16,21,22]. Furthermore, in our study, among seropositive participants, anti-HBs antibody levels differed across age groups. In the age group < 25 years, fewer participants were in the ≥100 IU/L category as compared to the other age groups. A possible explanation for this could be that the time elapsed since the last received vaccine dose was longer in younger individuals < 25 years, compared to individuals in the age groups ≥ 25 years, potentially due to a recently received booster dose, as it was previously shown that anti-HBs levels continuously decrease over time after having received an HBV vaccination [32]. However, as antibody levels of 10 IU/L or higher are assumed to be protective [8,9], there seems to be no particular relevance of having antibody levels of ≥100 IU/L. We also detected a higher anti-HBs seroprevalence in females as compared to males. A plausible explanation could be that, in Austria, more women than men are employed as healthcare workers and in care facilities, for whom booster vaccinations are recommended every 10 years and special vaccination campaigns have been implemented. It has also been shown that men are more likely than women to be non-responders to the HBV vaccine, defined as having an anti-HBs antibody level below 10 IU/L [33,34,35]. Additionally, our study showed a higher seroprevalence in urban areas than in rural areas among participants aged 40 to <55 years and ≥55 years. The lower seroprevalence among this age group living in rural areas may be due to limited access to vaccine information, greater distances to vaccination centers, higher levels of vaccine hesitancy [36,37], or a reduced willingness to travel in high HBV risk countries. Other studies on various vaccines have also found lower vaccination rates in rural areas compared to urban areas [37,38,39,40]. In summary, our data from a blood donor cohort in Tyrol, Austria, underline that HBV vaccination campaigns in younger people are effective, as in our study, seropositivity is strikingly higher in younger age groups where free vaccination programs were in place compared to older age groups. Further, our findings also support that catch-up vaccination programs, especially targeting the older age groups living in rural areas, are required to achieve higher HBV immunization rates and to close HBV immunity gaps in the general population of Austria. Our findings on seropositivity as well as anti-HBs antibody levels in different age groups are highly important, as there was no data available before our study was conducted, and now, conclusions can be drawn in order to perform specific vaccination campaigns to target particular subgroups of the population.

The study we presented herein has several important strengths. First, with data on 3935 participants, our study was adequately powered to reliably quantify overall seroprevalence as well as across population subgroups. Second, our findings are likely to be generalizable to age groups between 18 and 70 years, since blood donors represent a healthy sample of the adult general population. Our study also has some limitations. First, although individuals with a prior self-reported or acute HBV infection are excluded from blood donation, we cannot preclude a clear differentiation of infection- and vaccination-induced seropositivity because we have not measured anti-HBc antibodies, which indicate a former HBV infection at some point in their lives. Second, we were not able to collect information on vaccination dates due to time constraints at the blood donation centers. Third, with the data at hand, we cannot provide insight into the seroprevalences and antibody titers of groups not eligible for blood donation in Austria, as children, adolescents, people aged ≥ 70 years, and individuals with severe comorbidities. Fourth, we only analyzed data from Tyrol and not from other federal states in Austria.

As a future project, it would be interesting to analyze anti-HBc seroprevalence in blood donors in Tyrol in order to be able to clearly state how many people did unknowingly go through a hepatitis B infection and what proportion of the population immunity results from vaccination campaigns vs. from a previous infection. Further, it would also be relevant to analyze the dynamics of anti-HBs levels in more detail, which would be possible if vaccination dates of study participants could be collected and if the study cohort is followed up over a long period of time.

## 5. Conclusions

Seroprevalence of anti-HBs in blood donors from Tyrol, Austria, was 51.4% between August and September 2023 and differed across age, sex, and residence area. As anti-HBc antibodies are not required to be routinely measured in blood donors in Austria, we were not able to differentiate between vaccinated and recovered participants in our study cohort. However, catch-up vaccination programs, especially targeting the elderly living in rural areas, are needed to close HBV immunity gaps.

## Figures and Tables

**Table 1:** Characteristics of 3935 blood donors enrolled in our study.

Characteristic	No. (%), Median (25th–75th Percentile)
Date of assessment	5 September 2023 (28 August 2023–13 September 2023)
Age in years	47.6 (33.3–56.6)
Age group, years	
<25	475 (12.1%)
25 to <30	315 (8.0%)
30 to <35	299 (7.6%)
35 to <40	294 (7.5%)
40 to <45	401 (10.2%)
45 to <50	409 (10.4%)
50 to <55	550 (14.0%)
55 to <60	593 (15.1%)
60 to <65	400 (10.2%)
≥65	199 (5.1%)
Female sex	1603 (40.7%)
Prior pregnancy ^a^	998 (66.8%)
Urban area	1603 (40.7%)
Residence district	
Innsbruck-Stadt	171 (4.3%)
Innsbruck-Land	575 (14.6%)
Schwaz	509 (12.9%)
Kufstein	1056 (26.8%)
Kitzbühel	451 (11.5%)
Imst	350 (8.9%)
Landeck	629 (16.0%)
Reutte	194 (4.9%)
Anti-HBs, IU/L ^b^	539.3 [116.3–5417.0]
HBsAg	0 (0.0%)
HBV-DNA-PCR	0 (0.0%)

Missing values: 6.9% prior pregnancy, 6.7% HBV-DNA-PCR. Abbreviations: Anti-HBs, antibody to hepatitis B surface antigen; CI, confidence interval; HBsAg, hepatitis B surface antigen; HBV-DNA-PCR, hepatitis B virus DNA detection and quantification by real-time polymerase chain reaction; SD, standard deviation. ^a^ The percentage is quantified among 1493 females with non-missing data on pregnancy status. ^b^ The percentage is quantified among 2022 seropositive participants.

## Data Availability

Tabular data on the blood donor cohort can be requested from the corresponding authors by researchers who submit a methodologically sound proposal (including a statistical analysis plan); participant-level data on the blood donor cohort cannot be shared due to regulatory restrictions.

## Supplementary Materials

The following supporting information can be downloaded at: https://www.mdpi.com/article/10.3390/vaccines12101156/s1, Table S1: STROBE checklist. Reference [14] is cited in the Supplementary Materials.
